# Comparison of Local Anesthetic Effect of Bupivacaine versus Bupivacaine plus Dexamethasone in Nasal Surgery 

**Published:** 2013

**Authors:** Abdolhosein Ma’somi, Hasan Abshirini, Mahmood Hekmat shoar

**Affiliations:** 1 *Department of Otorhinolaryngology, Imam Khomeini Hospital, Ahwaz Jundishapur University of Medical Sciences, Ahwaz, Iran. *

**Keywords:** Analgesics, Anesthesia, Complications, Nasal Surgical Procedures, Pain

## Abstract

**Introduction:**

Adequate pain control is an important consideration in the post-surgical management of patients. Local nerve blockade added to general anesthesia can provide excellent pain control during and after most nasal surgical procedures. The aim of this study was to determine the combined effect of local anesthetic drugs with corticosteroids in nasal surgery.

**Materials and Methods:**

In this double-blind clinical study, 60 patients who underwent different nasal surgical procedures were matched and divided into two equal groups. Bilateral local nerve blockade was used in both groups. Bupivacaine or bupivacaine plus dexamethasone was administered by injection (groups B and B+D, respectively). Postoperative visual analog scale (VAS) pain values and the need for oral/intramuscular analgesic treatment in the first 24 h were recorded in all patients.

**Results:**

Thirty-eight male (63.3%) and 22 female (36.7%) patients were included in this study, with a mean age of 28.3 ± 8.2 years. At 1, 2, 4, 6, and 12 h post surgery, VAS pain values were significantly lower in the B+D group than in the B group. The analgesic requirement was significantly lower in the B+D group compared with the B group. No relevant complications were seen during surgery or postoperative hospitalization.

## Conclusion: 

This study demonstrates the positive effect of a combination of a dexamethasone with a bupivacaine in reducing pain and the need for analgesic drugs after different nasal surgeries. No acute or short-term post-surgical complications were observed in this study.

## Introduction

Appropriate pain control is an important consideration in the post-surgical management of patients. Physicians should ensure that therapeutic procedures are not the cause of extra and unnecessary pain to patients. Pain is particularly common after nasal surgery, especially when bone manipulation and periosteal irritation are involved. Substantial research and clinical observations suggest that pain reduction can be achieved in nasal surgery through use of local anesthesia(1).The use of local anesthesia in combination with general anesthesia is increasingly observed in head and neck surgery. In particular, local nerve blockade added to general anesthesia can provide excellent pain control during and after surgery in most nasal procedures (2,3).

Lidocaine and bupivacaine are commonly used for local injection or nerve blockade in nasal surgeries (4). 

Numerous studies performed in Iran and other countries have demonstrated an advantage of topical administration, local injection, and nerve blockade with bupivacaine or lidocaine versus saline. Other studies have shown an advantage of bupivacaine over lidocaine with regard to pain control and duration of pain control.

In this paper, we investigate the combined effect of dexamethasone with bupivacaine as local anesthesia after nasal surgery, in order to determine the optimal procedure for pain control and reduction of analgesic use post surgery. 

## Materials and Methods

This double-blind clinical study was carried out in the otorhinolaryngology ward of Imam-Khomeini Hospital, Ahwaz. A total of 60 patients who underwent different nasal surgical procedures (including closed nasal bone reduction, septoplasty, rhinoplasty and functional endoscopic sinus surgery (FESS)) under general anesthesia were matched according to demographic factors such as age, sex, BMI, type of surgery,…and divided into two groups of 30. Bilateral local nerve blockade of the infraorbital nerve, supratrochlear nerve, and terminal branches of the nasopalatine nerve was used in both groups immediately after general anesthesia.

Bupivacaine (Marcaine^®^; 0.5%, 5–20 cm^3^, with a mean dose of 1–1.5 mg/kg and a maximum dose of 100 mg) and bupivacaine plus dexamethasone (0.4%, 0.5–2 cm^3^, with a mean dose of 0.1 mg/kg) was administered by injection to the B and B+D groups, respectively. 

Visual analog scale (VAS) pain scores (0–100) were recorded 1, 2, 4, 6, 12, and 24 h postoperatively in all patients. A 325mg Acetaminophen (Paracetamol) tablet was available upon patient request postoperatively, with intramuscular Pethidine (Ampul 20mg) as ‘‘rescue’’ analgesia. The need for analgesic agents, including oral treatment or intramuscular (IM) injection in the first 24 h after the operation was recorded in a double-blinded manner. 

Post surgical pain and need to analgesics in the first 24 hours was Calculated and compared in any type of nasal surgery separately,and then final results were gathered together and Analysised Generally.

T-tests and chi-square tests were used for comparison of quantitative and qualitative variables. Final data were analyzed with descriptive statistics using with SPSS software (16^th^ Edition).

## Results

**Fig 1:**
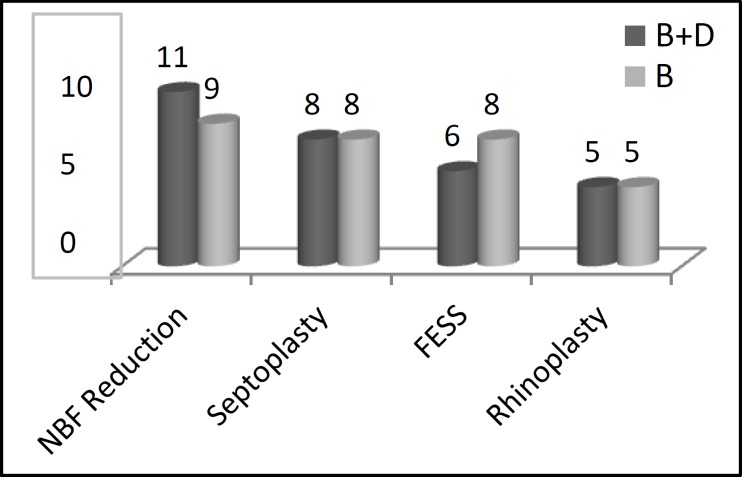
Different types of performed nasal surgery in B and B+D groups

Thirty-eight male (63.3%) and 22 female (36.7%) patients were included in this study, with a mean age of 28.3 ± 8.2 years. Patients underwent closed nasal bone reduction, septoplasty, FESS, and/or rhinoplasty (Fig 1). There were no significant differences between the groups with respect to age, gender, BMI, duration of operation, or type of surgery (Table 1). 

At 1, 2, 4, 6, and 12 h post surgery, VAS pain scores were significantly lower in the B+D group than in the B group(P<0.0001, P=0.002, P=0.023, P<0.0001 and P=0.011, respectively) but there was no statistically significant difference between the groups in VAS at 24 h after surgery P=0.221 (Table 2).

**Table 1:** Patients’demographic data

	**Group B+D**(n=30)	**Group** ** B**(n=30)
Age (Years)	29.8±7.3	26.8±9.1
Sex (M/F)	(20/10)	(18/12)
BMI (kg/cm2)	25.2±3.8	24.7±3.9mk
Main surgical duration (Min)	92.3±19	83.4±23

**Table 2:** Visual Analog Scale (VAS) pain scores at 1, 2, 4, 6, 12, and 24 h post surgery in 60 patients

**VAS Scores**	**Group B+D(n=30)**	**Group B** **(n=30)**	**P**
1 h post surgery	18.7±2.3	40.9±3.9	<0.0001
2 h post surgery	22.3±1.9	35.1±3.1	0.002
4 h post surgery	18.9±2.4	31.2±2.9	0.023
6 h post surgery	5.7±1.2	16.6±1.9	<0.0001
12 h post surgery	11.2±1.7	21.9±2.4	0.011
24 h post surgery	8.3±1.6	6.2±1.3	0.221

The analgesic requirement was significantly lower in the B+D group compared with the B group (P=0.038), with 12 and 18 patients requiring post-surgical oral/intra muscular analgesics, respectively (Table 3). No relevant complications (such as reduced blood pressure, bradycardia, visual problems, or hypersensitivity reactions) were observed during surgery or in the first 24 h of postoperative hospitalization.

**Table3:** Requirement for post-surgical analgesics in 60 patients

**Analgesic consumption**	**Group B+D** **(n=30)**	**Group** **B** **(n=30)**
Nil additional Analgesic	18	12
Oral Analgesic requirement	9	13
1 tablet (Acetaminophen 325 mg)	5	4
2 tablet (acetaminophen 325 mg)	2	4
3 tablet (acetaminophen 325 mg)	1	3
4 tablet (acetaminophen 325 mg)	1	2
Oral and intramuscular (20 mg pethidine) analgesic	3	5

## Discussion

Local anesthetic injection and nerve blockade have been used for many years, but the techniques are now more popular than ever. The major advantages of these techniques, whether used alone or adjacent to general anesthesia are their inherent simplicity and safety. Recently, studies have shown that local injection with bupivacaine (≤0.25% density) causes in local anesthesia and vasoconstriction (5).

The effect of different local anesthetic agents in comparison with normal saline and each other was reviewed in various studies. One study done by Edward and Colleagues in university of California demonstrated beneficial effect of bupivacaine versus normal saline in pain control after nasal surgery (6). This advantage for using of bupivacaine versus normal saline was proved in many other studies (7-9). 

Advantage of local injection with bupivacaine 0.25% in comparison with lidocaine 2%+ Epinephrine 1/100,000 in pain control and reduction need for post-surgical analgesics was shown In another study done by Yavuz and Colleagues in 2008 (10). This Finding also was seen in Yilmaz and Colleagues’ study (11).

In this investigation, no study was seen relative to combination corticosteroids with local anesthetic agents in Otorhin- olaryngologic fields but using this technics in other body areas was reported, as in study done by Kopacz and Colleagues in university of Seattle adding dexamethasone to bupivacaine for intercostal nerve blockade reduced time for local anesthetic's onset and prolonged painless and senseless duration significantly (12). In another study was done in university of Tehran in 2005, Doctor Movafegh and his cooperators pointed to positive effect of adding dexamethasone to lidocaine in lengthen duration of Brachial plexus nerve blockade (13). In our study, we observed a statistically significant benefit for the combination of bupivacaine and dexamethasone in terms of pain control and a reduced need for analgesic agents in the first 12 h after different nasal surgeries.

## Conclusion

The results from the present study demonstrate a positive benefit for the combination of dexamethasone with bupivacaine in the reduction of pain and requirement for analgesic drugs after different nasal surgical procedures. No acute or short-term post-surgical complications were observed in our study. Further larger studies seems to be inevitable in order to fully investigate these positive benefits.

## References

[1] Flint PW (2010). Cummings Otolaryngology Head and Neck Surgery.

[2] Flint PW (2010). Cummings Otolaryngology Head and Neck Surgery.

[3] Miller RD (2010). Miller’s Anesthesia.

[4] Miller RD (2010). Miller’s Anesthesia.

[5] Nahler G (2009). Dictionary of Pharmaceutical Medicine.

[6] Mariano ER, Watson D, Loland VJ, Chu LF, Cheng GS, Mehta SH (2009). Bilateral infraorbital nerve blocks decrease postoperative pain following nasal surgery. Can J Anesth.

[7] Leong P, Darvasula VSP, Boardman S, Back G (2003). Bupivacaine in nasal packs as topical analgesia after nasal surgery. International Congress Series.

[8] Mariano ER, Chu LF, Loland VJ, Cheng GS, Ilfeld BM (2008). Effect of infraorbital blocks on recovery duration following nasal surgery. Anesthesiology.

[9] Buchanan MA, Dunn GR, MacDougall GM (2005). Effect of topical bupivacaine on post-operative pain in bilateral nasal surgery. J Larygol Otol.

[10] Demiraran Y, Ozturk O, Guclu E, Iskender A, Ergin MH, Tokmak A (2008). Analgesic efficacy of locally infiltrated Levobupivacaine for nasal surgery. Anesth Analg.

[11] Yilmaz YF, Ozlage dik S, Titiz A, Tuncay A, Ozcan M, Unal A (2008). Comparison of Levo-bupivacaine and lidocaine for postoperative analgesia following septoplasty. Rhinol Journals.

[12] Kopacz DJ, Lacouture PG, Wu D, Nandy P, Swanton R, Landau C (2005). The dose response and effects of dexamethasone on bupivacaine for intercostal blockade in healthy volunteers. Anesth Analg.

[13] Movafegh A, Razazian M, Hajimohamadi F, Meysamie A (2006). Dexamethasone added to lidocaine prolongs axillarybrachial plexus blockade. Anesth Analg.

